# Visual suppression deficits as a biomarker of working memory impairment in schizophrenia

**DOI:** 10.1016/j.scog.2025.100395

**Published:** 2025-10-11

**Authors:** Cristina Filannino, Elliot Freeman, Andrew Parton, Neelam Laxhman, Corinna Haenschel

**Affiliations:** a Centre for Clinical, Social and Cognitive Neuroscience, Department of Psychology and Neuroscience, City St George's, University of London, London, UK; b Department of Psychology, Brunel University of London, UK; c East London NHS Trust, UK

**Keywords:** Schizophrenia, Working memory, Visual surround suppression, ERP

## Abstract

**Introduction:**

Although working memory (WM) deficits are well established in schizophrenia (SZ), their underlying source is still unclear. It has been proposed that these WM deficits may depend on an imbalance between cortical excitation and inhibition (E/I), but its importance for SZ remains unclear. A potential biomarker for E/I is visual Surround Suppression (SS), where the apparent contrast of a central grating is typically suppressed by a surround with parallel orientation (versus orthogonal). Here we exploited the SS phenomenon to test whether E/I contributes to WM impairments in schizophrenia.

**Methods:**

Using centre-surround gratings, we measured psychophysical thresholds for contrast matching, detection and orientation discrimination, in 21 SZ patients and 20 matched controls. Using the same stimuli, we also measured WM accuracy and event-related potentials (ERPs) in a delayed-match-to-sample task.

**Results:**

In SZ participants, reduced SS predicted impaired WM performance as well as general cognitive measures (CANTAB). Similar relationships were also observed between other early visual measures (impaired contrast detection and orientation discrimination), WM and general cognition. In response to SS, there was reduced amplitude visual ERPs (P1, N1 and P2) in patients compared with controls. Furthermore, across both groups the P1 amplitude correlated with visual SS.

**Conclusion:**

Together, these findings provide evidence that imbalances in cortical excitation and inhibition may contribute to visual and some cognitive deficits in schizophrenia, and that SS may provide a behavioural and electrophysiological biomarker.

## Highlights

• Visual surround suppression (SS) shows excitation/inhibition (E/I) imbalance in SZ.
• Impaired SS predicts working memory deficits in SZ.
• ERP correlate of SS found in controls, disrupted in SZ
• SS may provide a biomarker for visual and cognitive impairment.

## 1 Introduction

Working memory (WM) impairments are recognised as a core feature of schizophrenia (Forbes et al., 2009; Lawlor-Savage and Goghari, 2014; Park and Gooding, 2014; Lee and Park, 2005) that impact on quality of life and prognosis (Green et al., 2004, Green et al., 2000; Shamsi et al., 2011). Neurophysiological models have suggested that WM deficits in schizophrenia (SZ) may be explained by impaired cortical inhibition, which many researchers have linked to dysfunctions in gamma-aminobutyric acid (GABA) neurons (Lewis et al., 2004; Fish et al., 2022) or the imbalance between excitation and inhibition (Murray et al., 2014).

Research in schizophrenia has tended to focus on the effects of GABAergic abnormalities in higher cortical regions, such as prefrontal cortex. However, SZ-related reductions in GABA concentration have also been shown in other brain areas including the visual cortex (Yoon et al., 2010; Javitt, 2015; Qian et al., 2022). Measuring visual function in SZ therefore offers a convenient model for investigating the neurobiological differences underpinning SZ and its associated cognitive deficits. This is because the optimal stimuli for neurons in early visual cortex (for example V1) are well-characterised in comparison to those involved in higher level cognitive tasks; consequently, psychophysical procedures can be used to obtain precise measurements of visual function, which can be related more broadly to models of GABAergic imbalances and higher cognitive functions.

Previous studies have reported abnormalities in SZ for a range of low-level visual processing tasks including visual contrast sensitivity, orientation discrimination (Herrera et al., 2021; Shaw et al., 2020; Shoshina and Shelepin, 2015; Shoshina et al., 2021; Silverstein and Rosen, 2015). Importantly, abnormalities in inhibition have also been suggested as the basis for deficits in Surround Suppression (Yoon et al., 2010; Schallmo et al., 2018). In Surround Suppression (SS), a central target grating normally appears to have lower contrast when embedded in a larger stimulus sharing similar spatial properties, which facilitates lateral inhibitory activity between neurons in early visual processing areas (Blakemore and Tobin, 1972; Cannon and Fullenkamp, 1993; Xing and Heeger, 2001; Zenger-Landolt and Heeger, 2003; Hopf et al., 2006). Typically, SS is larger for ‘parallel’ surrounds, with a similar orientation to the central target, and smaller for ‘orthogonal’ surrounds, oriented at 90° to the target (Vanegas et al., 2015; Xing and Heeger, 2001). However, there is less susceptibility to this effect in SZ compared to controls (Barch et al., 2012; Dakin et al., 2005; Tibber et al., 2013; Yoon et al., 2009, Yoon et al., 2010), resulting in a more veridical perception of the central stimulus. This paradoxical SZ advantage may be related to the disruption of lateral inhibition in the primary visual cortex (Dakin et al., 2005; Seymour et al., 2013; Silverstein, 2016; Tibber et al., 2013; Yoon et al., 2009). Hence, SS has been suggested as a biomarker for the balance between neural excitation and inhibition in the visual cortex, but given the similarities in neuronal characteristics across the neocortex it may in principle be a more general biomarker (Douglas and Martin, 2004; Li et al., 2024).

Electrophysiological studies have investigated the sensory and higher-level WM-related responses to visual stimuli in healthy participants and SZ (Butler et al., 2008; Dias et al., 2011; Haenschel et al., 2007; Haenschel and Linden, 2011; Javitt, 2009; Park and Gooding, 2014). For example, the early visual P1 response from extrastriate cortex has been shown to reflect essential sensory characteristics such as contrast and luminance (Luck, 2014; Kosilo et al., 2022; Schindler et al., 2018). It has also been linked to effective WM encoding (Kosilo et al., 2022) and appears diminished in SZ (Haenschel et al., 2007). The P1 component has also been associated with attentional hyperfocus in SZ towards central versus peripheral stimuli (Kreither et al., 2017) which could affect stimulus encoding especially in the context of SS. While some of this evidence suggests that general early visual encoding deficits may explain WM dysfunction in SZ, ours is the first study to test specifically the extent to which visual ERP correlates of SS can account for WM disruption in SZ.

With respect to SS specifically, smaller early visual ERPs have been reported to parallel compared to orthogonal surrounds in healthy participants (Joo and Murray, 2014; Haynes et al., 2003; Nguyen et al., 2020), but in SZ results on contextual effects are mixed. One study reported enhanced orientation-dependence of suppression in the N1 component in schizophrenia (Klein et al., 2025), alongside weakened orientation-insensitive suppression of P1; in contrast another study found a weakened suppression of context for contour stimuli in SZ was only related with P2 (Pokorny et al., 2021), a component linked to attention and feature detection (Luck and Hillyard, 1994). The present study used SS to test whether any disruptions of neural suppression at the level of P1, N1 or P2 are related to WM performance deficits in SZ.

Here, we examined the interaction between WM and SS using psychophysical and EEG methods. We examined the behavioural and event-related potentials (ERPs) responses to a delayed-matched-to-sample WM task using centre-surround stimuli designed to evoke SS. In a set of psychophysical experiments, we also obtained psychophysical measures of SS, orientation discrimination and contrast detection thresholds. Finally, we correlated our measures with performance on spatial working memory (SWM) and paired associate learning (PAL) subtasks of the CANTAB battery.

We hypothesized that reduced Surround Suppression would be associated with reduced WM accuracy in SZ. We tested whether WM accuracy also correlated with impairment in basic visual function as measured by orientation discrimination and contrast detection thresholds. We also aimed to measure the electrophysiological correlate of Surround Suppression, relate the magnitude of such effects to the psychophysical measure of SS, and to WM performance. Finally, we tested to what extent higher cognitive functions measured in the CANTAB battery could be predicted by the WM task, visual performance, and the EEG correlates.

## 2 Methods

### 2.1 Participants

Twenty-three clinically stable and medicated people with SZ (ICD-10 criteria) and 20 healthy control participants (CT) matched in terms of gender, age and years of education were recruited (see Table 1 for demographics). All participants had normal or corrected to normal vision. The study was approved by an NHS Research Ethics Committee (14/LO/1535).

**Table 1:** Participants' demographic details and SZ's clinical characteristics.

Characteristics	SZ (*N* = 21)	Controls (*N* = 20)
Mean age (SD)	36.3 (9.9)	34.95 (10.75)
Male	13	12
Female	8	8
Mean (SD) years of education	13 (2.2)	13.4 (1.8)
Handedness^a^		
Right	20	20
Left	1	
Mean (SD) years since diagnosis	4.55 (3.3)	
Mean (SD) PANSS Score	20 (4.6)	
CPZ (SD)	295.7 (298.11)	

PANSS: Positive and Negative Syndrome Scale (Kay et al., 1987). CPZ: Chlorpromazine equivalent. 21 SZ were medicated; 3 with Amisulpride, 5 with Aripiprazole, 4 with Clozapine, 4 with Olanzapine, 2 with Quetiapine, 2 with Risperidone and 1 with Risperidone-DEPOT.

a Edinburgh Handedness Inventory Questionnaire (Oldfield, 1971).

Two SZ showed chance-level and random responses in all tasks and were excluded from the analysis. The final sample consisted of 21 SZ and 20 Controls. Average Chlorpromazine (CPZ) equivalent (mean CPZ: 295.7) was calculated per each patient based on the chlorpromazine equivalents (CPZ) published conversion factors (Leucht et al., 2016).

### 2.2 Stimuli

**Fig. 1:**
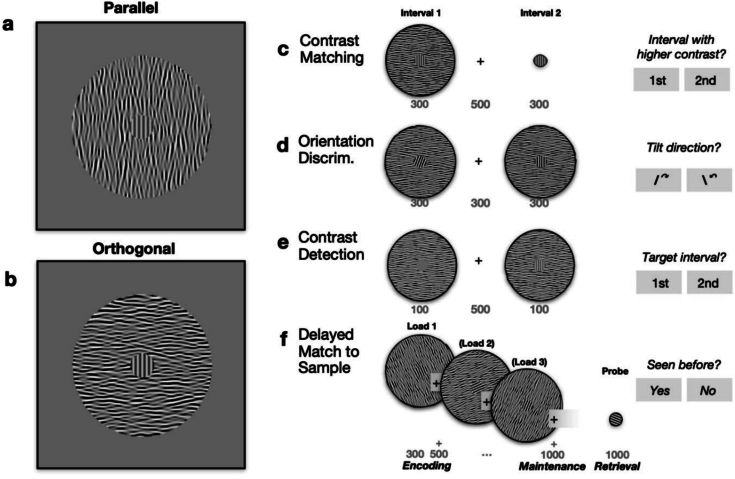
Stimuli and tasks. (a–b) Central target gratings with parallel and orthogonal surrounds. Maximum surround suppression of the Target contrast is expected with parallel surrounds. Contrast has been enhanced for illustration purposes. (c–f) Trial sequence for four tasks. Grey numbers show durations of stimulus displays and inter-stimulus intervals (+) in milliseconds.

Stimuli for all tasks comprised a circular Target grating (radius 0.67 degrees of visual angle, spatial frequency 4 cycles/degree) embedded in a larger circular Surround of radius 4 degrees, composed of bandpass filtered white noise (4 cycles/degree, bandwidth one octave), sampling orientations over a range of ±15°. The orientation of the surround relative to the target was either 0° (‘Parallel’, Fig. 1a) or 90° (‘Orthogonal’, Fig. 1b). Michelson contrast for the surround was always 100 % throughout tasks and trials, but varied for the target according to the task.

### 2.3 Design and procedure

We obtained psychophysical measures of visual performance in this order: contrast detection threshold in the two surround conditions (Parallel versus Orthogonal) (Fig. 1e), matching contrast between target stimuli with versus without a surround (Fig. 1c), and orientation discrimination threshold as a test of basic stimulus visibility (Fig. 1d). Each participant's average contrast detection threshold was multiplied by a factor of 15 to set the supra-threshold contrast levels in the main WM match-to-sample task (Fig. 1f), from which we obtained a measure of proportion correct (accuracy). EEG was recorded only during the WM task.

#### 2.3.1 Contrast matching (CM)

To measure SS, participants compared the contrast of a Target grating presented in two consecutive intervals (Fig. 1c). Target contrast was fixed at 30 % in the second interval and served as reference. A Parallel or Orthogonal surround at maximum contrast was only present in the first interval. Participants pressed the left or right arrow on a keyboard to indicate the interval in which the target appeared to have higher contrast. Matching contrast was estimated using an adaptive method of adjustment. A matching contrast that is higher than the reference contrast indicates suppression of perceived contrast, such that greater physical contrast is required to achieve a subjective match. Surround suppression (SS) can then be calculated by subtracting matching contrast for Parallel surrounds from Orthogonal, such that negative values indicate greater SS.

#### 2.3.2 Delayed matching to sample working memory task

The WM load was manipulated by presenting one, two or three target gratings with the surround for 300 ms each, with an ISI of 500 ms (encoding phase, Fig. 1f) and a maintenance period of 1000 ms. Finally, a probe grating without the surround was presented for 1000 ms (retrieval phase). Participants had to press the left or right arrow on a keyboard to indicate if the orientation of the probe matched (or not) any of the orientations of the gratings presented during the encoding phase.

#### 2.3.3 ERP data acquisition, processing and analysis

Recording, digitization, and pre-processing of the EEG data were performed with a BrainAmp amplifier and the BrainVision Recorder software (Brain Products, Munich, Germany). The EEG was recorded at a sampling rate of 500 Hz with a system bandpass between 0 and 100 Hz. A 64-channel electrode cap was fitted to the participants' head with an additional vertical electro-oculogram electrode below the left eye. Electrode impedance was kept below 20 kΩ.

EEG analysis during encoding was performed with BrainVision Analyser software (Brain Products GmbH). EEG data for correct trials were segmented into intervals between 200 ms before and 1000 ms after stimulus onset and baseline corrected from -200 ms to stimulus onset. Electrodes were chosen based on Schallmo et al. (2019) and Kosilo et al. (2022) and confirmed by visual inspection of the grand averages. For the P1 and N1, we averaged across electrodes PO7, PO8, PO9 & PO10 and for P2 we chose Oz, O1 and O2.

#### 2.3.4 Cambridge Neuropsychological Test Automated Battery (CANTAB)

Two CANTAB tests were used to assess visuospatial and working memory abilities (Barnett et al., 2010; Sahakian et al., 1988). In the Paired Associate Learning (PAL) test, participants match patterns to previously seen locations to assess visual memory and new learning. In the Spatial Working Memory (SWM) test, participants search an array of hidden locations for tokens, without returning to previously searched locations to assess recall and manipulation of spatial information in WM (Owen et al., 1990). Lower scores indicate a more optimal strategy.

## 3 Results

### 3.1 Behavioural results

#### 3.1.1 Working memory task

**Fig. 2:**
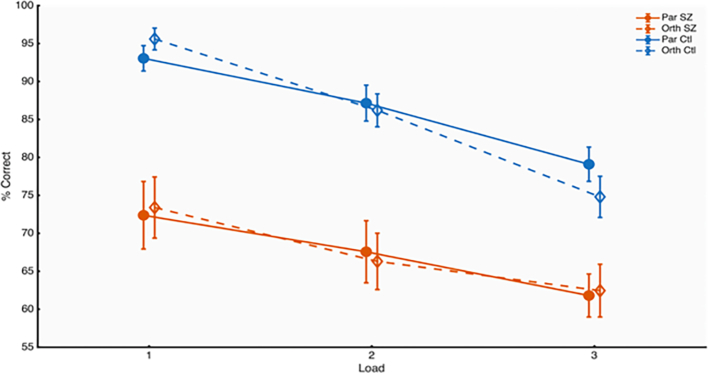
Working memory accuracy as a function of load, split by participant group, SZ (red), control (blue). Separate lines for parallel (Par, continuous lines) and orthogonal surround (Orth, dotted lines). Error bars indicate one unit of standard error.

Mixed ANOVA showed that accuracy was significantly lower in SZ (Fig. 2) [F(1,78) = 21.26, *p* < 0.0001, pη^2^ = 0.35] and decreased with increasing Load [F(2,78) = 74.73, p < 0.0001, pη^2^ = 0.66]. There was a significant Load x Group interaction [F(2,78) = 4.46, *p* = 0.0147, pη^2^ = 0.10], showing a reduced effect of Load in SZ.

#### 3.1.2 Visual tasks

**Fig. 3:**
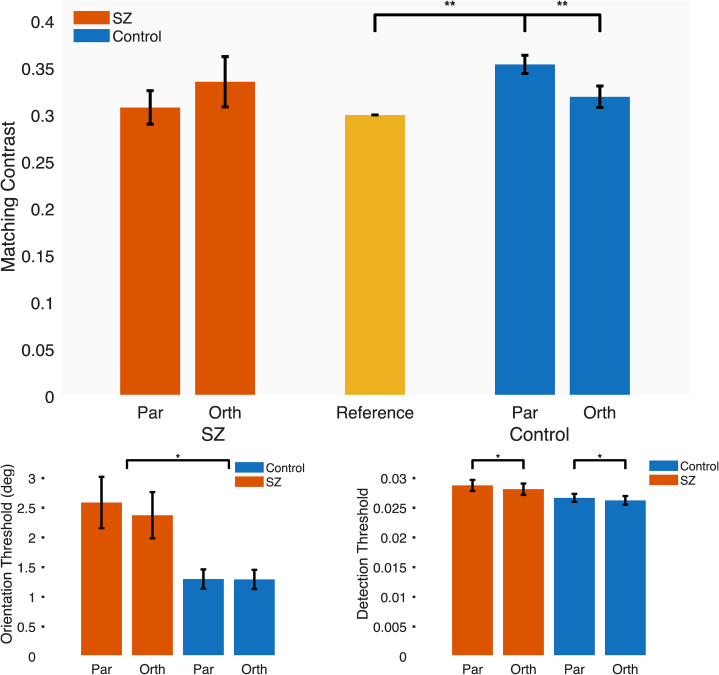
Top: Matching contrasts for central gratings in the context of parallel versus orthogonal surrounds. Higher values indicate that the apparent contrast of the central grating was suppressed so that its physical contrast had to be adjusted higher to perceptually match the fixed reference. Separate bars are shown for SZ (red) and control groups (blue). Error bars denote one unit of standard error. Lower left: Orientation thresholds for gratings with parallel vs orthogonal surrounds, split by SZ and control groups, with standard error bars. Lower right: Contrast detection thresholds for gratings with parallel vs orthogonal surrounds, split by SZ and control groups, with standard error bars.

For Contrast Matching, mixed ANOVA showed that the Surround x Group interaction was significant [F(1,39) = 4.68, *p* = 0.04, pη^2^ = 0.107]. There were no other significant effects. Post-hoc tests showed that matching contrast was on average significantly higher with parallel surrounds compared to orthogonal in Controls [Parallel vs Orthogonal t(19) = 3.47, *p* = 0.003, Cohen's D = 0.73], and compared to the Reference [t(19) = 5.53, *p* < 0.001, Cohen's D = 1.75], but for SZ these comparisons were not significant [*p* > 0.2] (Fig. 3, top).

For Orientation discrimination (Fig. 3, lower left), there was a significant main effect only of group [F(1,32) = 10.89, *p* = 0.0024, pη^2^ = 0.25], where mean discrimination thresholds were significantly higher in SZ (mean 2.48 degrees, SE 0.29) compared to Controls (mean 1.30, SE 0.11).

For Contrast Detection (Fig. 3, lower right), there was a significant main effect only of surround [F(1,39) = 7.12, p = 0.01, pη^2^ = 0.16], where detection thresholds were significantly higher for Parallel surrounds (mean 0.0277, SE 0.0006) compared to Orthogonal (mean 0.0272, SE 0.0006).

#### 3.1.3 Correlations between visual tasks and working memory accuracy

**Fig. 4:**
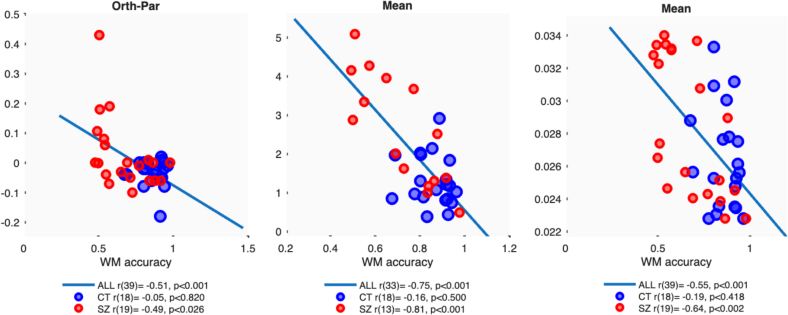
Top row: Scatterplots of contrast matching (left), orientation thresholds (middle) and surround suppression (orthogonal minus parallel, right), against average working memory performance. Separate colours and Pearson's r correlation statistics are shown for control (blue markers) and SZ (red markers) with a line of best fit shown.

For the contrast matching task, we calculated the Surround Suppression effect by subtracting matching contrast for gratings with Parallel surrounds from the Orthogonal condition. Negative values indicate greater Surround Suppression from Parallel Surrounds, which is the typical pattern. A significant negative correlation with WM accuracy was observed across the whole sample [p < 0.001] (Fig. 4, left). However, within groups the correlation was only significant for SZ [*p* < 0.026], where lower accuracy was associated with a reduction or even reversal of the SS effect. Correlations differed significantly between SZ and Control [Fisher's Z = 2.07, *p* = 0.038].

Orientation Discrimination thresholds correlated negatively with WM accuracy for both surround configurations, but just for SZ [Parallel *p* < 0.002, Orthogonal *p* < 0.006, Mean p < 0.001] (Fig. 4, middle). These correlations differed significantly from the non-significant correlations found in the Control group [e.g. for the Mean data Fisher's Z = 4.23, *p* = 0.00002]. Similar results were observed for Contrast Detection thresholds [Parallel p < 0.001, Orthogonal p < 0.006, Mean p < 0.002, Fisher's Z = 2.42, *p* = 0.015] (Fig. 4, right).

There was no correlation between chlorpromazine equivalents and contrast matching, orientation discrimination, contrast detection and WM accuracy in line with previous studies (Foxe et al., 2001; Haenschel et al., 2007; Sawaki et al., 2018; Silverstein and Rosen, 2015).

### 3.2 ERP results

**Fig. 5:**
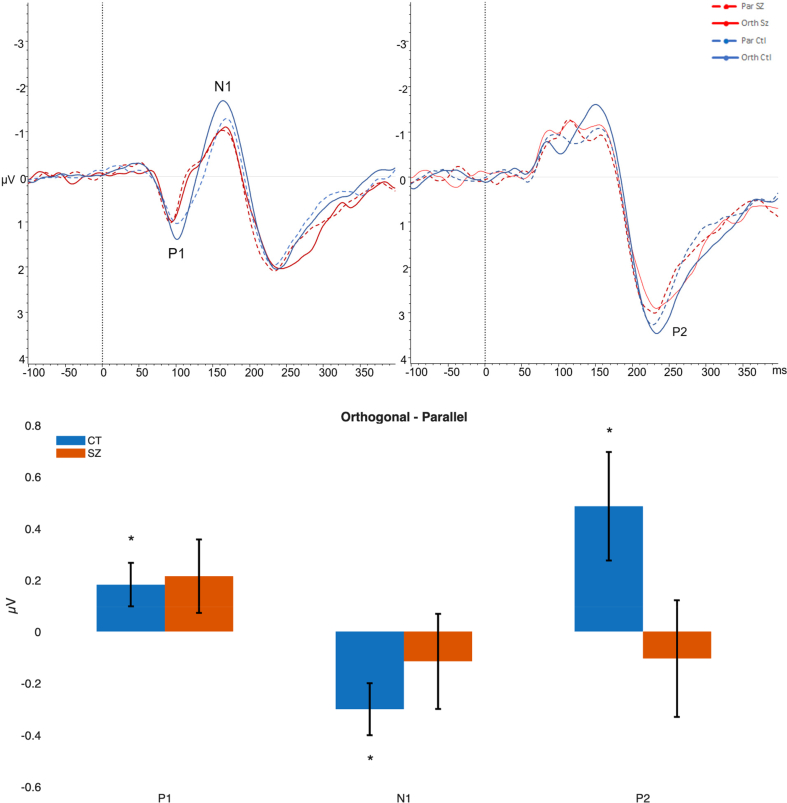
Top grand average ERPs during WM encoding, for parallel (dotted lines) and orthogonal conditions (continuous lines), for SZ (red lines) and controls (blue lines) across PO7, PO8, PO9, PO10 (left) and O1, OZ, O2 (right). Time on the x-axis is shown in milliseconds. Bottom: Average peak ERP amplitude (μV) for orthogonal surrounds after subtracting parallel. Error bars indicate one standard error.

P1 and N1 were observed at lateral occipital electrodes (PO7, PO8, PO9, PO10) with a mean latency of 105 ms (SD = 24) for SZ, and 103 ms (SD = 19) for Controls and 158 ms (SD = 27 ms) for SZ and 163 ms (SD = 26 ms) for Controls, respectively (Fig. 5). P2 was elicited at central visual electrodes (Oz, O1 and O2) with a mean latency of 230 ms (SD = 23 ms) for SZ and 232 ms (SD = 20 ms) for Controls (Fig. 5, top).

A mixed ANOVA analysing ERPs during the stimulus encoding phase showed significant main effects for Component [F(2,78) = 119.36, *p* < 0.0001, pη^2^ = 0.75] and Memory Load [F(2,78) = 13.95, p < 0.0001, pη^2^ = 0.26], where ERP amplitudes were on average greatest for the minimum load condition. Load did not interact with any other variables.

There was a significant interaction between ERP Component, Surround Orientation (Parallel vs Orthogonal), and participant group [F(2,78) = 3.50, *p* = 0.035, pη^2^ = 0.08]. Simple effects analyses examined this interaction effect, after subtracting amplitudes for Parallel surrounds from Orthogonal. For each component the orthogonal response was significantly greater than for parallel for Control participants [*p* < 0.05], but these comparisons were not significant for SZ (Fig. 5, bottom). The difference in amplitudes appeared most prominent for P2, however between-group comparisons for individual components were not significant.

#### 3.2.1 Correlations

**Fig. 6:**
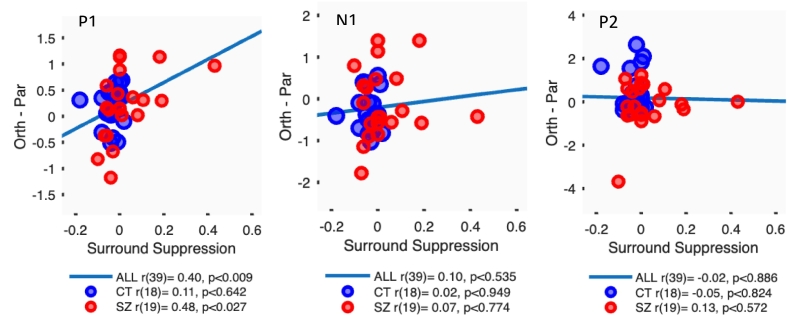
Scatterplots of the three ERP components (P1, N1, and P2, in μV) showing orthogonal minus parallel surrounds against surround suppression (contrast matching for orthogonal – parallel surrounds).

For the P1 component, greater Surround Suppression (higher perceived contrast of gratings in orthogonal relative to parallel surrounds) was correlated with more positive P1 amplitudes for Orthogonal relative to Parallel surrounds, across the whole sample [*p* < 0.009]. This pattern was significant within the SZ group [*p* < 0.027] but not the control group (Fig. 6, left) [Fisher's Z = 1.81, *p* < 0.07]. This effect depended specifically on the response to Orthogonal surrounds, where lower matching contrast (i.e. higher perceived contrast) was associated with higher P1 amplitudes, across the whole sample [*p* < 0.005], and for SZ [*p* < 0.03] specifically. There was a similar weaker trend in N1 for Orthogonal stimuli, across the whole sample [*p* < 0.026] (Fig. 6, middle). There were no significant trends for Parallel surrounds in either component. In contrast, for P2, the Control sample showed a significant positive correlation between matching contrast and P2 amplitudes for Parallel surrounds [p < 0.02] but not the Orthogonal surrounds [Fisher's Z = 2.25, *p* = 0.02], and not for SZ (Fig. 6, right). The form of this correlation is consistent with an effect of Parallel surrounds suppressing apparent contrast (and raising matching contrast) more in Controls, while surround suppression is reduced in SZ. There were no significant correlations between average ERPs and average Orientation thresholds or Detection thresholds. There were also no significant correlations between ERP peak amplitudes and WM accuracy. There was no effect of CPZ equivalent effects across the ERPs analysis.

### 3.3 CANTAB

SZ showed significantly higher error scores than controls in Paired Associate Learning [t(39) = −2.92, *p* < 0.006, Cohen's D = 0.9] and in Spatial Working Memory [t(39) = −2.22, p < 0.03, Cohen's D = 0.70], however there was no significant difference in scores for Spatial Working Memory strategy. All CANTAB scores correlated significantly with accuracy in the Working Memory task, for SZ only.

Correlation analyses showed that WM accuracy significantly predicted all three CANTAB measures in SZ, but not Controls; SS, Orientation discrimination and Contrast Discrimination all significantly predicted PAL and SWM errors across the whole sample (all p < 0.05).

## 4 Discussion

We examined the relationship between WM and early visual processing mechanisms in participants with schizophrenia compared with healthy controls. SZ participants showed a reduction, or even reversal, of the normally suppressive effect of a surround in the contrast matching task and their accuracy in this task was correlated with WM performance, but not for control participants. Furthermore, contrast detection thresholds and orientation discrimination thresholds also predicted lower WM performance in SZ but not in controls.

There were also differences in the ERP components in people with SZ when compared with healthy controls. For each ERP component (P1, N1 and P2), the perceived contrast in the orthogonal condition was significantly greater than for parallel condition for control participants, but not significant for participants with SZ. This indicated a strong surround suppression effect in the healthy controls but not SZ groups. Interestingly, across both groups, the P1 amplitudes in response to orthogonal compared to parallel stimuli in the WM task were proportional to perceived contrast (or the strength of surround suppression).

Our results revealed a general pattern of basic visual deficits in schizophrenia, which are associated with subsequent impairment in WM performance. More specifically, we replicated findings of reduced contextual processing in SZ (Butler et al., 2008; Dakin et al., 2005; Barch et al., 2012) as well as finding deficits in contrast detection and orientation discrimination (Tibber et al., 2013; Pokorny et al., 2023). All of these deficits were associated with reduced WM accuracy (Haenschel et al., 2007; Gold et al., 2020, Gold and Luck, 2023). Taken together, broader and weaker representations in the visual system (see also Qian et al., 2022) may have specifically affected the efficiency and/or precision with which visual stimuli are encoded into WM, leading to impaired encoding (Park and Gooding, 2014) and making them consequently harder to recall (Murray et al., 2014). The correlation between P1 during WM encoding and surround suppression across the sample suggests that higher neural suppression enables better early visual encoding across the sample. Interestingly, the larger amplitudes across all visual ERPs in response to orthogonal compared with parallel conditions may support the notion that inhibitory neuronal characteristics can be found across early and higher (visual) areas (Li et al., 2024).

Surround suppression has been used as marker for neural suppression in early visual areas (Javitt, 2009; Phillips and Silverstein, 2013). Previous studies have demonstrated a range of abnormalities in the excitatory/inhibitory balance on visual processing (Qian et al., 2022; van Loon et al., 2016) as well as WM performance (Fish et al., 2022; Lewis et al., 2004; Koychev et al., 2017; Murray et al., 2014). For example, we previously found reduced WM performance following ketamine, a NMDA receptor blocker (Koychev et al., 2017) on a delayed matching to sample task similar to reduced WM performance in SZ (Haenschel et al., 2007). Reduced WM performance was associated with an increase in early P1 amplitude. The increase in P1 was interpreted as loss of lateral NMDA modulation contributing to cortical disinhibition. This may suggest that similar mechanisms across the visual and WM excitatory/inhibitory network may contribute to these abnormalities in SZ. This is in line with Li et al.'s (2024) suggestion that abnormalities in circuitry mechanisms and excitation/inhibition balance may be involved in both surround suppression and cognitive dysfunction. Importantly, although its functional role varies across cortical areas, NMDA has been proposed to underlie dysfunctional processes in SZ at all processing levels (Phillips and Silverstein, 2013). As a consequence, developing sensitive behavioural and neurophysiological tools for measuring its function could form the basis for a biomarker for SZ and allow monitoring of its progression (Schielke and Krekelberg, 2021). Though further research is needed, we would argue that surround suppression may provide this window into changes in NMDA function. In support of this, a recent study by Schielke and Krekelberg (2021) showed reduced processing of context in a contrast illusion (the Chubb illusion) in Macaques following administration of ketamine, which has been shown to be dependent on the NMDA system.

Future studies will need to further investigate the neural mechanisms underlying the relationship between SS and WM. In addition, it is possible that WM performance was disrupted by abnormal control of attention in SZ (Bansal et al., 2020; Hahn et al., 2020; Luck and Gold, 2008, Luck et al., 2019a, Luck et al., 2019b), reduced target salience (Mayer et al., 2014, Mayer et al., 2018) or attentional lapses (Barch et al., 2012). Barch et al. (2012) showed that attentional lapses may contribute to SS in schizophrenia, which may have also had an effect on WM encoding. In line with Gold and Luck (2023), future research needs to disentangle the effect of SS and WM abnormalities during encoding while controlling for attentional lapses.

While our study focused on the impact of surround inhibition on encoding, there may be a further important role for neural suppression during maintenance/delay period (Kiyonaga and Egner, 2016), which may also be disrupted in SZ (Bansal et al., 2020; Gold et al., 2020). Thus, abnormal neural suppression effects may vary depending on the paradigm, and future studies need to disentangle its contribution to the different phases of WM.

Our finding that people with schizophrenia made more CANTAB PAL and SWM errors compared to controls is in line with previous evidence (Wannan et al., 2018; Levaux et al., 2007). In the SZ group, number of PAL and SWM errors also correlated negatively with accuracy in our WM task, which suggests that reduced visual memory performance in SZ was related to WM performance and that this generalises to well established neurocognitive measures of visual spatial and short-term memory. Both CANTAB PAL and SWM also correlated with surround suppression across the whole sample, which provides a further indication for the potential use of SS in the visual cortex as a biomarker for higher cognitive deficits.

Finally, some limitations need to be considered. First, our sample size was relatively small and as such the findings need to be interpreted as exploratory and not confirmatory. Future research needs to replicate our findings in a larger group. Because of the number of tests and to avoid fatigue, the number of trials in the adaptive staircase was relatively small. More trials would have ensured the procedure to determine the thresholds to be more stable. Despite these limitations, this study is the first to our knowledge to examine the relationship between neural suppression and WM in people with schizophrenia.

This study has shown that SZ is associated with impairment of early visual processes, including disrupted SS measured both perceptually and electrophysiologically. These early visual deficits are predictive of higher cognitive tasks, i.e. WM performance. Our study contributes to the understanding of how visual processes interact with WM and future research should address the extent to which similar abnormalities in inhibition and/or excitation in individuals with schizophrenia contribute to cognitive impairments and identify biomarkers for the disease progression.

## CRediT authorship contribution statement

**Cristina Filannino:** Writing – review & editing, Writing – original draft, Methodology, Investigation, Formal analysis, Conceptualization. **Elliot Freeman:** Writing – review & editing, Writing – original draft, Supervision, Software, Methodology, Formal analysis, Conceptualization. **Andrew Parton:** Writing – review & editing, Conceptualization. **Neelam Laxhman:** Investigation. **Corinna Haenschel:** Writing – review & editing, Writing – original draft, Supervision, Resources, Project administration, Methodology, Funding acquisition, Formal analysis, Conceptualization.

## Declaration of Generative AI and AI-assisted technologies in the writing process

During the preparation of this work the author(s) used no AI or AI-assisted technologies in the writing process.

## Declaration of competing interest

The authors have nothing to declare.
